# Investigation the interaction of dietary fat quality indices and the MC4R gene in metabolically healthy and unhealthy overweight and obese women

**DOI:** 10.1038/s41598-023-38988-9

**Published:** 2023-07-27

**Authors:** Niloufar Rasaei, Melika Fallah, Mohammad Nemati, Fatemeh Gholami, Rasool Ghaffarian-Ensaf, Khadijeh Mirzaei

**Affiliations:** 1 Department of Community Nutrition, School of NutritionalSciences and Dietetics, Tehran University of Medical Sciences (TUMS), P.O. Box: 14155-6117, Tehran, Iran; 2 Department of Nutrition, Science and Research Branch, Islamic Azad University, Tehran, Iran

**Keywords:** Genetics, Obesity

## Abstract

Obesity has become a common global problem. Some obese people can be metabolically healthy. Gene-environment interaction can be important in this context. This study aimed to assess the interaction between dietary fat quality indices and the Melanocortin 4 receptor (MC4R) gene in metabolically healthy and unhealthy overweight and obese women. This cross-sectional study was conducted on 279 women with overweight and obesity. The definition of metabolically healthy and unhealthy phenotypes was done according to Karelis criteria. Dietary assessment was done using a 147-item validated semi-quantitative food frequency questionnaire and dietary fat quality was assessed by cholesterol-saturated fat index (CSI) and the ratio of omega-6/omega-3 (N6/N3) essential fatty acids. MC4R was genotyped by polymerase chain reaction-restriction fragment length polymorphism technique. A generalized linear model was used to evaluate the interaction between dietary fat quality indices and the MC4R gene in both crude and adjusted models. Study subjects with higher ratio of N6/N3 had higher homeostatic model assessment for insulin resistance (HOMA IR) index (*P* = 0.03) and other variables showed no difference according to the tertile of CSI and N6/N3. Participants with the C allele of MC4R rs17782313 had lower height (*P* < 0.001) and higher HOMA index (*P* = 0.01). We found that the CC genotype of MC4R interacts with the N6/N3 ratio on the metabolically unhealthy phenotype in the crude model (β = 9.94, CI 2.49–17.39, *P* = 0.009) and even after adjustment for all confounders (β = 9.002, CI 1.15–16.85, *P* = 0.02, β =  − 12.12, CI 2.79–21.46, *P* = 0.01). The data of this study can justify one inconsistency observed in society, regarding dietary recommendations about metabolic health status. Those with CC genotype, are more likely to have an unhealthy phenotype with an increase in N6/N3 as one fat quality indices than those who do not have CC genotype. We found the interaction of dietary fat quality indices such as N6/N3 and the MC4R gene in metabolically unhealthy overweight and obese women.

## Introduction

Obesity has become a very important health issue worldwide and its prevalence has increased dramatically, becoming an uncontrollable epidemic^1,2^. Complications of obesity, such as cardiovascular diseases, diabetes, and cancer can cause serious health problems for people and high costs to the health care system^3,4^. Obese people have different phenotypes with different metabolic risks^5^. A group of obese people who do not have metabolic complications is considered metabolically healthy obese (MHO) people^6,7^. Meanwhile, obese individuals with obesity-related metabolic complications are known as metabolically unhealthy obese (MUO)^8^. Obesity is a multifactorial phenomenon in which genetic and environmental factors (such as diet) and their interaction can affect its occurrence^1^. So far, many genes have been associated with the risk of obesity such as Iroquois homeobox protein 3 (IRX3) and retinitis pigmentosa GTPase regulator-interacting protein 1 like (RPGRIP1L)^9^. Therefore, gene-environment interaction can be considered one of the most important determinants of obesity risk^10,11^.

The existence of diverse findings can probably be attributed to changes in the genetic background of individuals and gene-diet interactions^12,13^. Variation in the melanocortin-4 receptor (MC4R) gene is known as the most common genetic cause of obesity^14^. Variants of MC4R-rs17782313 can affect food intake, total energy intake, fat intake, appetite, and consequently the occurrence of obesity^15,16^. On the other hand, protective effects on obesity have also been seen in some variants in the MC4R gene^17^. MC4R rs17782313 has three types of genotypes: CC, TC, and TT. The relationship between these genotypes and obesity and related factors has been investigated in some studies. Findings of a cross-sectional study showed interactions between CC genotype and high stress, high appetite, and high energy and fat intake are likely associated with higher BMI. As a result, it seems that people with C allele of MC4R rs17782313 are more susceptible to overweight or obesity^18^. A study conducted in Asia showed that the minor allele C can be associated with a 1.55 times increase in the risk of obesity, while the homozygous CC genotype can have a stronger effect and is associated with a 2.43 times increase in the risk of obesity in women^19^.

Results of a cross-sectional study showed that participants with low-frequency alleles of MC4R rs17782313 had a greater risk of metabolically unhealthy obesity^20^. In addition, the result of a previous study showed that the probability of metabolically healthy obesity was higher in patients with the T/C genotype of MC4R rs17782313^21^. Therefore, genetic variants in MC4R rs17782313 are considered as an important factor to understand the cause and type of obesity phenotypes^22^.

Studies have shown that total dietary fat intake is related to the risk of obesity^23,24^. However, today there is more focus on the quality of consumed fat than its quantity^25^. To investigate the effect of type of fat intake, previous studies have suggested dietary fat quality indices, such as cholesterol-saturated fat index (CSI) and the ratio of omega-6 to omega-3 (N6/N3). CSI was introduced in 1986 by Connor et al.^26^ and N6/N3 was proposed by Simopoulos et al.^27^. Findings regarding the relationship between the type of dietary fat and the risk of obesity are inconsistent. Several studies have shown a positive association between saturated fat intake and obesity^28,29^, while the opposite finding has been observed in studies^30,31^. Also, regarding the consumption of polyunsaturated fatty acid (PUFA), positive^23^, negative^32^, and null^33^ associations with obesity have been observed. In a cross-sectional study, an inverse relationship between N6/N3 ratio and general and abdominal obesity was seen^34^. Also, results of a study showed that subjects with metabolically unhealthy obesity had a higher intake of n-6/n-3 PUFA ratio than metabolically healthy obese person^35^. In addition, the results of a study showed a positive association between CSI and one of the risk markers of cardiovascular diseases in overweight and obese people^36^.

Despite the importance of investigating the interaction of genes and diet, which can help make dietary recommendations more specific to people, human studies in this field are limited and inconclusive. In this cross-sectional study, we aimed to assess the interaction between dietary fat quality indices and the MC4R gene in metabolically healthy and unhealthy overweight and obese women.

## Materials and methods

### Study population

The participants of this cross-sectional study were overweight and obese women. 279 women from the health centers of Tehran University of Medical Sciences were included according to the inclusion criteria. Body mass index (BMI) of 25–40 kg/m^2^ and age range of 18–68 were the inclusion criteria. We excluded participants who had the following conditions: history of malignancies, acute or chronic diseases, cardiovascular disease, all types of diabetes, thyroid disease, renal or hepatic disease, taking drugs to lower blood pressure, sugar and blood lipids, taking weight loss supplements or using a specific diet in the past year, smoking and presence of pregnancy, lactation or menopause. All methods were carried out in accordance with relevant guidelines and regulations. All participants completed written informed consent. Also, the ethics committee of the TUMS approved the study protocol. (Ethics number: IR.TUMS.MEDICINE.REC.1401.498).

### Anthropometric and blood pressure measurements

Weight, body mass index, fat-free mass (FFM), and body fat (BF) percentage were measured by a bioelectrical impedance analyzer (BIA) (InBody 770 scanner from InBody Co. (Seoul, Korea)) according to the manufacturer’s protocols^37^. Participants had to take off their shoes, sweaters, and coats and not have metal objects such as earrings, rings, and watches with them. Height was measured in a standing position without shoes using a non-elastic tape with an accuracy of 0.5 cm. Regarding the waist circumference (WC) and hip circumference (HR) of participants, the narrowest part of the waist (after expiration) and the largest part of the hip were measured respectively using an elastic tape with an accuracy of 0.5 cm. Then waist to hip ratio (WHR) was calculated by the formula.

### Definition of metabolically healthy and unhealthy phenotypes

Karelis criteria^38^ were used to classify participants in terms of metabolic health. In this way, 5 items were examined: (1) Triglycerides ⩽ 1.7 mmol/L, (2) Low-density lipoprotein (LDL) ⩽ 2.6 mmol/L and no treatment, (3) High-density lipoprotein (HDL) ⩾ 1.3 mmol/L and no treatment, (4) C-reactive protein (CRP) ⩽ 3.0 mg/L and 5) homeostatic model assessment for insulin resistance (HOMA-IR) ⩽ 2.7. If ⩾ 4 items were present, the individual was considered metabolically healthy.

### Physical activity assessment

The physical activity (PA) of the participants in the last week was assessed by the reliable and validated International Physical Activity Questionnaire-short form (IPAQ) and measured in terms of metabolic equivalent hours per week (METs-h/week)^39^.

### Biochemical and hormonal determination

Biochemical evaluations were carried out in the Nutrition and Biochemistry Laboratory of the School of Nutritional and Dietetics, TUMS. After 10–12 h of fasting, serum samples were collected. First samples were centrifuged, stored at − 80 °C, and analyzed using a single assay technique. Triglyceride (TG) was assayed using glycerol-3-phosphate oxidase–phenol 4-amino antipyrine peroxidase (GPOPAP) enzymatic endpoint^40^. Also, high-density lipoprotein (HDL) and low-density lipoprotein (LDL) cholesterol were evaluated by direct enzymatic clearance assay^41^. High sensitive C reactive protein (hs-CRP) level was assessed by immunoturbidimetric assay. Randox Laboratories (Hitachi 902) kits were used for all assessments. The level of insulin was measured and HOMA-IR was calculated according to this formula: HOMA-IR = insulin (Mu/mL) × fasting glucose (mmol/L)/22.5^42^.

### Dietary intake assessment

Assessment of the participants’ dietary intake in the last year was done using a 147-item validated semi-quantitative standard food frequency questionnaire (FFQ)^43^. Individuals were asked to report the consumption frequency of each food item on a daily, weekly, monthly, or yearly basis and the questionnaire was completed by an expert dietician. The intake of macronutrients, micronutrients, and total energy were analyzed by the NUTRITIONIST 4 (First Data Bank, San Bruno, CA) food analyzer^44^.

### Dietary fat quality indices

Dietary fat quality was assessed by two indices: (1) cholesterol-saturated fat index (CSI) and (2) the ratio of N6/N3 essential fatty acids. Food intakes extracted from FFQ were used to calculate both of these indices. CSI was calculated according to this formula CSI = (1.01 × g saturated fat) + (0.05 × mg cholesterol)^26^. N6/N3 was calculated by dividing N6/N3 contents of foods^27^.

### The Genotype determination

The polymerase chain reaction-restriction fragment length polymorphism (PCR–RFLP) technique was used for genotyping MC4R rs17782313 and rs1333048 single-nucleotide polymorphisms (SNPs) (genotypes C&T).

To extract MC4R Genomic deoxyribonucleic acid (DNA), we used 200 mL of whole blood and the GeneAll Mini Columns Type kit (GeneAll, South Korea). Extracted DNA, was used to evaluation of 2 SNPs reported near the MC4R gene, rs17782313, and rs17700633 SNP, which was performed. Using a specific primer (89 forward primer 5AAGTTCTACCTACCATGTTCTTGG-3 and reverse primer 5-90TTCCCCCTGAAGCTTTTCTTGTCATTTTGAT-3), polymerase chain reaction (PCR) was performed on these SNPs. PCR was performed using 50 ng/ml DNA, 10 mmol of each primer, and 1 M dimethyl sulfide (total volume equal to 20 μl). The amplification steps included the following items, respectively: primary denaturation at 94 °C (5 min), 35 cycles of denaturation at 60 °C (1 min), annealing at 94 °C (45 s), extension at 72 °C (1 min), and final extension at 72 °C (10 min). The SNPs rs7041 (Thr, 420, Lys) and rs4588 (Asp, 416, Glu) were auscultated by StyI and HaeIII enzymes according to the following procedure: The StyI enzymes (1μL) and HaeIII enzymes (1μL) each one separately added to the PCR product (5μL), distilled water (D.W.) (8μL) and Buffer Y. Tango (10 × 1μL). Products obtained from the digestion process were stained (with ethidium bromide) on a 2% agarose gel and imaging was performed. To confirm PCRFLP results, 10% of the sample were sequenced directly. Sequencing was performed using an ABI PRISM 3730 automated sequencer (Applied Biosystems, Foster City, CA, USA)^45^, and as a result, fragments containing 3 genotypes, CC, CT, and TT, were distinguished.

### Statistical analyses

The normality distribution of data was checked by Kolmogorov–Smirnov test. Demographic characteristics of individuals were presented as mean ± standard deviation, minimum and maximum. One-way analysis of variance (ANOVA) was performed to compare anthropometric indices, lipid profile, hs-CRP level, insulin and HOMA-IR between individuals. In order to eliminate the effect of confounding factors, analysis of covariance (ANCOVA) was performed. Post-hoc multiple comparison analysis (Bonferroni corrected) was employed to look into the mean differences between the groups. A generalized linear model (GLM) was used to evaluate the interaction between dietary fat quality indices and MC4R gene in both crude and adjusted models. The results of the analyzes were adjusted for BMI, age, physical activity and energy intake. SPSS version 23.0 (SPSS, Chicago, IL, USA) was used for data analyzed. *P*-value < 0.05 was considered statistically significant and interaction *P*-value < 0.1 was determined as mariginally significant.

### Ethics approval and consent to participate

This study was supported by grants from the Tehran University of Medical Sciences (TUMS), Tehran, Iran. Each individual was informed completely regarding the study protocol and provided a written and informed consent form before taking part in the study.

## Results

### Descriptive characteristics of the study sample

The current study included 279 women who were either overweight or obese. Individuals' age, weight, BMI, CSI, and N6 per N3 mean and standard deviation (SD) were 36.84 ± 8.45 years, 79.99 ± 10.88 kg, 30.73 ± 3.72 kg/m2, 12.65 ± 5.29, and 12.65 ± 0.10 respectively. Among the genotypes of 279 obese women with the MC4R gene, 40.9% of participants had TT, 26.2% TC and 33% had CC genotypes.

### General characteristics of study population according to tertiles of CSI and N6/N3 in obese and overweight women

**Figure 1:**
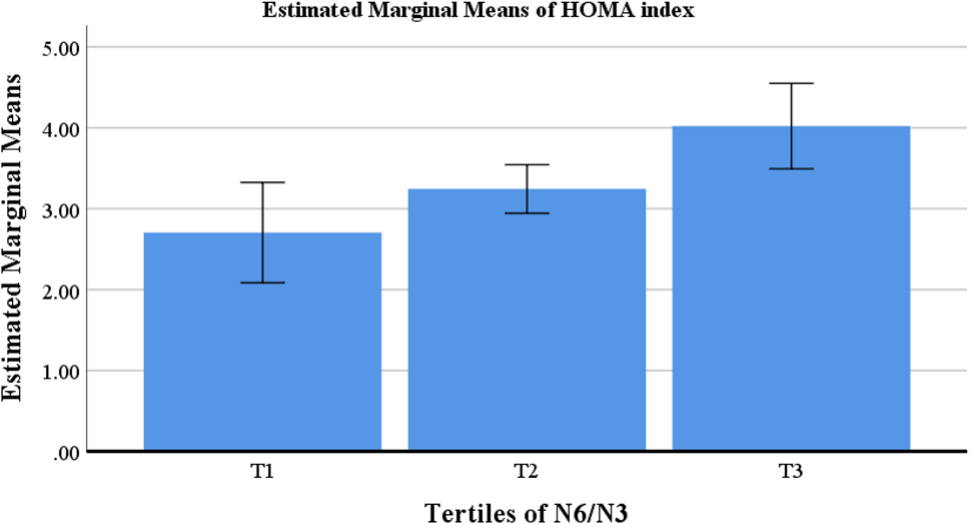
Estimated marginal means of HOMA index between tertiles of N6/N3 in obese and overweight women (n = 279).

Table 1 shows the key characteristics of the study population concerning the tertile categories of CSI and N6/N3 in obese and overweight women. Before adjusting for confounders, the results displayed a significant difference across the CSI category for age (*P* = 0.02) which disappeared after adjustment (*P* = 0.27). Other variables showed no association with the tertiles of CSI before and after adjustment. No variables had a significant association with N6/N3 tertiles in the crude model, but after controlling for confounding factors, a higher HOMA index was associated with a higher N6/N3 (*P* = 0.03) (Table 1). Figure 1 shows the estimated marginal means for HOMA index to identify between which tertiles of N6/N3 the differences could be found.

**Table 1:** General characteristics of study population according to tertiles of CSI and N6/N3 in obese and overweight women (n = 279).

Variables†	CSI				
	Mean ± SD			*P*-value	*P*-value*
	T_1_ (n = 99)	T_2_ (n = 104)	T_3_ (n = 76)		
Age (years)	37.97 ± 8.31	36.51 ± 8.23	34.48 ± 8.64	**0.02**	0.27
Anthropometric measurements					
Weight (kg)	78.78 ± 9.91	80.58 ± 11.53	80.76 ± 11.19	0.38	0.74^¥^
Height (cm)	160.57 ± 5.94	161.58 ± 5.69	161.94 ± 5.82	0.25	0.89^¥^
WC (cm)	97.45 ± 8.49	98.83 ± 9.80	99.07 ± 9.67	0.44	0.83^¥^
WHR (ratio)	0.92 ± 0.047	0.93 ± 0.054	0.93 ± 0.051	0.41	0.80^¥^
BMI (kg/m^2^)	30.62 ± 3.54	30.80 ± 3.79	30.77 ± 3.89	0.94	0.97^¥^
VFL (cm^2^)	17.16 ± 19.87	16.78 ± 13.49	15.67 ± 3.37	0.78	0.49^¥^
FFMI	17.82 ± 1.43	19.24 ± 12.86	17.80 ± 1.41	0.35	0.45^¥^
FMI	12.89 ± 2.99	12.89 ± 2.94	12.96 ± 3.06	0.98	0.92^¥^
Biochemical variables					
TC (mmol/l)	4.77 ± 0.807	4.76 ± 1.02	4.69 ± 0.93	0.85	0.66
TG (mmol/l)	1.39 ± 0.91	1.40 ± 0.79	1.27 ± 0.55	0.57	0.68
HDL (mmol/l)	1.22 ± 0.26	1.20 ± 0.31	1.19 ± 0.21	0.72	0.87
LDL (mmol/l)	2.45 ± 0.59	2.40 ± 0.65	2.44 ± 0.60	0.81	0.38
IPAQ (MET min-week)	855.11 ± 1067.64	1113.51 ± 1190.64	1003.86 ± 961.72	0.29	0.45
HOMA index	3.42 ± 1.40	3.17 ± 1.17	3.48 ± 1.27	0.26	0.42
hs.CRP (mg/l)	3.75 ± 4.31	3.99 ± 4.31	5.06 ± 5.29	0.20	0.23
Education%(n)				0.20	–
Illiterate	3 (3)	0 (0)	0.0 (0)		
Primary education	46 (6)	30.8 (4)	23.1 (3)		
Intermediate education	52.9 (9)	23.5(4)	23.5(4)		
High school education	57.1 (4)	14.3 (1)	28.6 (2)		
Diploma	32.1 (26)	43.2 (35)	24.7 (20)		
Postgraduate education	48 (12)	28 (7)	24 (6)		
Bachelor's degree and higher	29.3 (39)	39.8 (53)	30.8 (41)		
Marriage%(n)				0.33	–
Married	35.9 (78)	36.9 (80)	27.2 (59)		
Single	35.2 (19)	37 (20)	27.8 (15)		
Away from spouse more than 6 month	0.0 (0)	100.0 (1)	0.0 (0)		
Dead spouse	0.0 (0)	0.0 (0)	100.0 (2)		
Divorce	40 (2)	60(3)	0.0 (0)		
Met healthy%(n)				0.90	–
MH	45.3 (29)	37.5 (24)	17.2 (11)		
MUH	32.7 (55)	37.5 (63)	29.8 (50)		

Variables†	N6/N3				
	Mean ± SD			*P*-value	*P*-value*
	T_1_ (n = 93)	T_2_ (n = 93)	T_3_ (n = 93)		
Age (years)	35.95 ± 8.20	36.08 ± 8.45	37.40 ± 8.72	0.43	0.29
Anthropometric measurements					
Weight (kg)	81.12 ± 10.74	80.84 ± 11.89	78.01 ± 9.77	0.09	0.37^¥^
Height (cm)	162.02 ± 5.47	161.79 ± 5.77	160.15 ± 6.09	0.05	0.72^¥^
WC (cm)	98.81 ± 9.13	99.62 ± 10.11	96.79 ± 8.49	0.10	0.18^¥^
WHR (ratio)	0.92 ± 0.047	0.94 ± 0.054	0.92 ± 0.049	0.07	0.14^¥^
BMI (kg/m^2^)	30.90 ± 3.93	30.91 ± 3.63	30.37 ± 3.61	0.53	0.46^¥^
VFL (cm^2^)	15.58 ± 3.32	19.06 ± 24.55	15.20 ± 3.14	0.13	0.07^¥^
FFMI	17.91 ± 1.35	19.47 ± 13.52	17.63 ± 1.41	0.23	0.43^¥^
FMI	13.02 ± 3.14	12.86 ± 2.86	12.84 ± 2.97	0.90	0.92^¥^
Biochemical variables					
TC (mmol/l)	4.61 ± 0.75	4.77 ± 0.97	4.85 ± 1.01	0.26	0.10
TG (mmol/l)	1.33 ± 0.76	1.36 ± 0.81	1.39 ± 0.79	0.88	0.23
HDL (mmol/l)	1.19 ± 0.26	1.22 ± 0.28	1.20 ± 0.27	0.72	0.81
LDL (mmol/l)	2.40 ± 0.52	2.44 ± 0.64	2.45 ± 0.66	0.83	0.89
IPAQ (MET min-week)	960.36 ± 926.07	1192.29 ± 1445.85	812.75 ± 727.60	0.08	0.14
HOMA index	3.22 ± 1.27^a^	3.23 ± 1.27^b,a^	3.54 ± 1.30^c^	0.19	**0.03**
hs.CRP (mg/l)	4.64 ± 4.80	3.97 ± 4.61	3.98 ± 4.43	0.59	0.07
Education%(n)				0.58	–
Illiterate	0.0 (0)	66.7 (2)	33.3 (1)		
Primary education	30.8 (4)	53.8 (7)	15.4 (2)		
Intermediate education	35.3 (6)	23.5 (4)	41.2 (7)		
High school education	28.6 (2)	42.9 (3)	28.6 (2)		
Diploma	37.0 (30)	32.1 (26)	30.9 (25)		
Postgraduate education	16 (4)	40 (10)	44 (11)		
Bachelor’s degree and higher	35.3 (47)	30.8 (41)	33.8 (45)		
Marriage%(n)				0.59	–
Married	32.7 (71)	33.6 (73)	33.6 (73)		
Single	33.3 (18)	33.3 (18)	33.3 (18)		
Away from spouse more than 6 month	0.0 (0)	0.0 (0)	100.0 (1)		
Dead spouse	100.0 (2)	0.0 (0)	0.0 (0)		
Divorce	40.0 (2)	40.0 (2)	20.0 (1)		
Met healthy%(n)				0.82	–
MH	29.7 (19)	32.8 (21)	37.5 (24)		
MUH	33.9 (57)	31 (52)	35.1 (59)		

*BMI* body mass index, *FFM* fat free mass, *HDL* high density lipoprotein, *HOMA* homeostatic model assessment, *hs-CRP* high-sensitivity C-reactive protein, *SD* standard deviation, *T* tertile, *TC* total cholesterol, *TG* triglyceride, *VFL* visceral fat level, *MH* metabolic healthy, *MUH* metabolic unhealthy, *IPAQ* international physical activity questionnaires, *FFMI* fat-free mass index, *FMI* fat mass index, *WC* waist circumference, *WHR* waist to hip ratio.

Values are represented as means (SD).

Categorical variables: % (n).

†Calculated by analysis of variance (ANOVA).

*P*-value*: ANCOVA was performed to adjusted potential confounding factors (age, BMI, energy intake, physical activity).

¥: BMI consider as a collinear variable for anthropometric measurements and these variables adjusted for Age, physical activity, and total energy intake.

*p* < 0.05 was considered significant.

Values in the same row with different superscript letters are significantly different.

Significant values are in bold.

### General characteristics of study population according to MC4R rs17782313 in obese and overweight women

**Figure 2:**
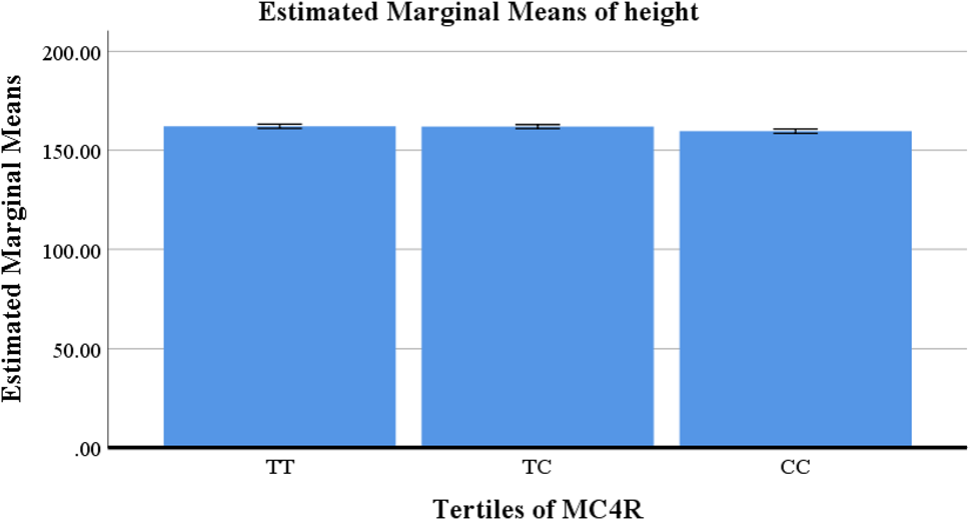
Estimated marginal means of height between tertiles of MC4R in obese and overweight women (n = 279).

**Figure 3:**
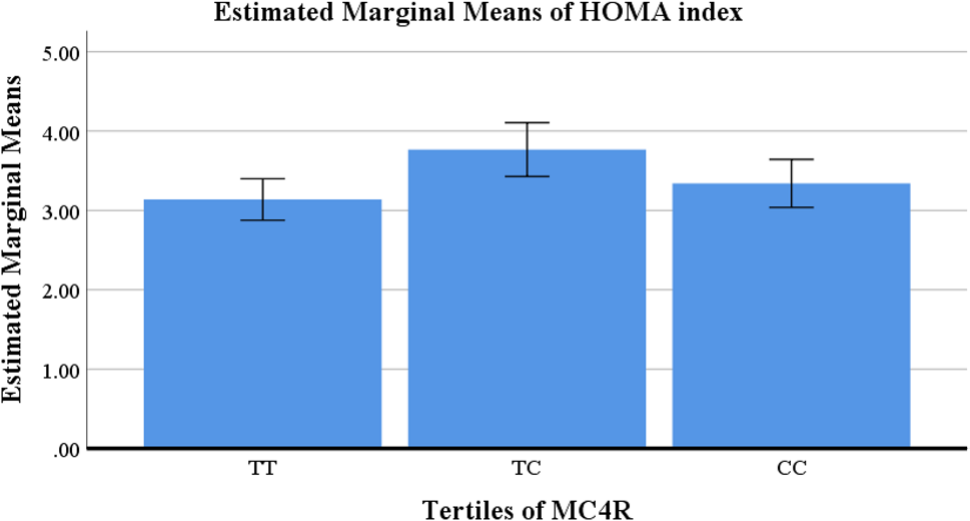
Estimated marginal means of HOMA index between tertiles of MC4R in obese and overweight women (n = 279).

There were no significant differences in anthropometric measurements and biochemical variables in crude models, but the C allele carrier of MC4R showed lower height (*P* =  < 0.001) and higher HOMA index (*P* = 0.01) after adjustment (Table 2). Figures 2 and 3 shows the estimated marginal means for height and HOMA index, respectively to identify between which tertiles of MC4R the differences could be found.

**Table 2:** General characteristics of study population according to MC4R rs17782313tertiles of MC4R in obese and overweight women (n = 279).

Variables†	MC4R				
	Mean ± SD			*P*-value	*P*-value*
	TT = 114	TC = 73	CC = 92		
Age (years)	36.78 ± 8.09	36.64 ± 9.45	35.98 ± 8.12	**0.78**	0.37
Anthropometric measurements					
Weight (kg)	80.49 ± 11.43	79.34 ± 10.06	79.89 ± 10.90	0.77	0.21^¥^
Height (cm)	161.83 ± 5.95^a^	161.14 ± 5.55^b,a^	160.83 ± 5.88^c^	0.44	** < 0.001**^¥^
WC (cm)	98.90 ± 9.91	97.93 ± 8.60	98.19 ± 9.15	0.76	0.25^¥^
WHR	0.93 ± 0.05	0.93 ± 0.04	0.92 ± 0.04	0.25	0.08^¥^
BMI (kg/m^2^)	30.77 ± 3.79	30.53 ± 3.60	30.83 ± 3.76	0.87	0.87^¥^
VFL (cm^2^)	18.33 ± 22.15	15.31 ± 3.01	15.48 ± 3.24	0.25	0.22^¥^
FFMI	17.95 ± 1.44	17.86 ± 1.45	19.20 ± 13.69	0.44	0.41^¥^
FMI	12.92 ± 3.09	12.67 ± 2.64	13.09 ± 3.12	0.67	0.64
Biochemical variables					
TC (mmol/l)	4.71 ± 0.88	4.92 ± 1.05	4.65 ± 0.86	0.21	0.53
TG (mmol/l)	0.89 ± 0.089	0.76 ± 0.099	0.63 ± 0.072	0.41	0.58
HDL (mmol/l)	1.16 ± 0.26	1.24 ± 0.29	1.22 ± 0.27	0.16	0.05
LDL (mmol/l)	2.37 ± 0.58	2.52 ± 0.66	2.45 ± 0.61	0.32	0.24
IPAQ (MET min-week)	1047.60 ± 1147.35	1000.15 ± 939.60	912.08 ± 1129.44	0.71	0.32
N6 per N3	12.66 ± 0.10	12.65 ± 0.11	12.63 ± 0.10	0.17	0.36
CSI	12.13 ± 4.73	13.19 ± 6.04	12.85 ± 5.31	0.36	0.44
HOMA index	3.13 ± 1.09^a^	3.71 ± 1.56^b^	3.33 ± 1.23^c,a,b^	0.25	**0.01**
hs.CRP (mg/l)	4.22 ± 4.67	3.87 ± 4.59	4.40 ± 4.56	0.80	0.33

*BMI* body mass index, *FFM* fat free mass, *HDL* high density lipoprotein, *HOMA* homeostatic model assessment, *hs-CRP* high-sensitivity C-reactive protein, *SD* standard deviation, *T* tertile, *TC* total cholesterol, *TG* triglyceride, *VFL* visceral fat level, *IPAQ* international physical activity questionnaires, *FFMI* fat-free mass index, *FMI* fat mass index, *WC* waist circumference, *WHR* waist to hip ratio.

Values are represented as means (SD).

†Calculated by analysis of variance (ANOVA).

*P*-value*: ANCOVA was performed to adjusted potential confounding factors (age, BMI, energy intake, Physical activity).

¥: BMI consider as a collinear variable for anthropometric measurements and these variables adjusted for Age, physical activity, and total energy intake.

*p* < 0.05 was considered significant.

Values in the same row with different superscript letters are significantly different.

Significant values are in bold.

### Dietary intake of study population according to tertiles of CSI and N6/N3 in obese and overweight women

Higher CSI was linked to more intake of refined grains, vegetables, fish, poultry, egg, red meat, protein, carbohydrate, total fat, cholesterol, PUFA, saturated fatty acid (SFA), linolenic acid, Eicosapentaenoic acid (EPA), and docosahexaenoic acid (DHA). Across the higher tertile of N6/N3, we found lower consumption of carbohydrates, monounsaturated fatty acid (MUFA), PUFA, oleic acid, and linoleic acid (Table 3).

**Table 3:** Dietary intake of study population according to tertiles of CSI and N6/N3 in obese and overweight women (n = 279).

Variables†	CSI			
	Mean ± SD			*p*-value*
	T_1_ (n = 99)	T_2_ (n = 104)	T_3_ (n = 73)	
Food group				
Whole grains (g/d)	53.24 ± 47.75	61.59 ± 54.19	77.42 ± 73.40	0.82
Refined grains (g/d)	331.80 ± 219.09^a^	379.17 ± 191.55^b,a^	397.52 ± 219.72^c^	**0.01**
Nuts (g/d)	9.45 ± 10.99	14.59 ± 15.63	21.40 ± 20.64	0.25
Legumes (g/d)	42.47 ± 34.57	51.43 ± 42.42	46.40 ± 42.31	0.27
Vegetables (g/d)	288.84 ± 183.46^a^	424.89 ± 241.68^b^	445.77 ± 262.62^c,b^	**0.003**
Fruits (g/d)	388.93 ± 312.69	510.63 ± 334.57	648.63 ± 34.94	0.92
Fish (g/d)	7.06 ± 6.33^a^	12.59 ± 12.24^b^	15.62 ± 15.97^c,b^	** < 0.001**
Poultry (g/d)	23.03 ± 18.74^a^	35.25 ± 26.63^b,a^	50.75 ± 62.74^c^	**0.002**
Egg (g/d)	12.60 ± 7.02^a^	21.47 ± 9.40^b^	33.85 ± 17.9^c^	** < 0.001**
Red meat (g/d)	12.20 ± 8.51^a^	22.38 ± 16.72^b^	32.84 ± 23.24^c^	** < 0.001**
Nutrient intake				
Energy (kcal/d)	2136.53 ± 601.19	2650.61 ± 674.27	3151.67 ± 612.17	–
Protein (g/d)	66.53 ± 17.27^a^	91.41 ± 20.92^b^	112.36 ± 28.38^c^	** < 0.001**
Carbohydrate (g/d)	305.72 ± 102.04^a^	385.51 ± 120.18^b,a^	435.97 ± 96.75^c^	** < 0.001**
Total fat (g/d)	78.52 ± 29.67^a^	91.87 ± 26.72^b,a^	116.48 ± 30.52^c,a^	**0.03**
Cholesterol (g/d)	305.72 ± 102.04^a^	385.51 ± 120.18^b^	435.97 ± 96.75^c^	** < 0.001**
MUFA (g/d)	27.06 ± 11.69	29.95 ± 8.87	37.72 ± 10.50	0.05
PUFA (g/d)	18.96 ± 10.07^a^	19.46 ± 7.35^b^	21.64 ± 7.44^c,b^	** < 0.001**
SFA (mg/d)	20.80 ± 6.43^a^	26.59 ± 6.44^b,a^	39.04 ± 12.35^c^	** < 0.001**
Trans fatty acid	0.0007 ± 0.002	0.0005 ± 0.001	0.001 ± 0.004	0.05
Oleic acid (g/d)	24.86 ± 11.50	26.86 ± 8.71	33.18 ± 9.97	0.06
Linolenic acid (g/d)	1.03 ± 0.66	1.19 ± 0.54	1.50 ± 0.61	0.46
Linoleic acid (g/d)	16.85 ± 9.56^a^	16.76 ± 7.10^b^	18.13 ± 7.14^c,b^	** < 0.001**
EPA (g/d)	0.01 ± 0.02^a^	0.03 ± 0.03^b^	0.04 ± 0.04^c,b^	** < 0.001**
DHA (g/d)	0.06 ± 0.06^a^	0.11 ± 0.12^b^	0.14 ± 0.13^c,b^	** < 0.001**

Variables†	N6/N3			
	Mean ± SD			*P*-value*
	T1(n = 93)	T2(n = 93)	T3(n = 93)	
Food group				
Whole grains (g/d)	76.88 ± 67.78	70.52 ± 59.97	41.42 ± 38.36	0.17
Refined grains (g/d)	489.62 ± 239.45	340.17 ± 194.30	272.29 ± 117.29	0.46
Nuts (g/d)	21.11 ± 19.00	15.81 ± 17.75	6.95 ± 6.07	0.36
Legumes (g/d)	51.82 ± 40.69	52.32 ± 44.80	36.50 ± 31.08	0.18
Vegetables (g/d)	439.80 ± 243.54	417.86 ± 256.50	289.23 ± 183.76	0.06
Fruits (g/d)	750.11 ± 382.63	439.53 ± 243.73	325.48 ± 209.00	0.46
Fish (g/d)	13.75 ± 15.65	11.24 ± 11.07	9.36 ± 8.81	0.99
Poultry (g/d)	45.60 ± 55.96	31.70 ± 29.99	28.12 ± 23.10	0.32
Egg (g/d)	12.6 ± 7.02	21.47 ± 9.40	33.85 ± 17.9	0.38
Red meat (g/d)	31.64 ± 20.16	20.75 ± 19.16	12.47 ± 8.39	0.05
Nutrient intake				
Energy (kcal/d)	3468.72 ± 402.67	2545.52 ± 190.36	1799.81 ± 271.01	
Protein (g/d)	114.98 ± 24.09	87.51 ± 17.49	62.37 ± 13.30	0.58
Carbohydrate (g/d)	502.95 ± 82.83	353.96 ± 47.13	255.92 ± 53.31	0.09
Total fat (g/d)	122.50 ± 27.88	95.28 ± 20.53	63.74 ± 15.19	0.09
Cholesterol (g/d)	502.95 ± 82.83	353.96 ± 47.13	255.92 ± 53.31	0.43
MUFA (g/d)	39.10 ± 9.87^a^	32.22 ± 9.23^b,a^	21.80 ± 6.55^c,a,b^	**0.03**
PUFA (g/d)	24.25 ± 7.54^a^	21.12 ± 8.80^b,a^	14.24 ± 5.48^c,a,b^	**0.02**
SFA (mg/d)	37.54 ± 11.27	27.37 ± 6.58	18.86 ± 5.14	0.38
Trans fat	0.001 ± 0.002	0.0007 ± 0.002	0.0008 ± 0.003	0.60
Oleic acid (g/d)	34.87 ± 9.55^a^	29.18 ± 9.32^b,a^	19.55 ± 6.46^c,a,b^	**0.02**
Linolenic acid (g/d)	1.58 ± 0.55	1.26 ± 0.67	0.82 ± 0.40	0.07
Linoleic acid (g/d)	20.80 ± 7.42^a^	18.44 ± 8.59^b,a^	12.27 ± 5.34^c,a,b^	**0.03**
EPA (g/d)	0.03 ± 0.04	0.03 ± 0.04	0.02 ± 0.02	0.83
DHA (g/d)	0.12 ± 0.13	0.10 ± 0.12	0.08 ± 0.08	0.94

*CSI* cholesterol to saturated fat index, *DHA* docosahexaenoic acid, *EPA* eicosapentaenoic acid, *MUFA* monounsaturated fatty acid, *PUFA* polyunsaturated fatty acid, *SFA* saturated fatty acid, *T* tertile, *TC* total cholesterol.

Data are mean ± SD.

*P*-value*: ANCOVA was performed to adjust the potential confounding factor (energy intake).

*p* < 0.05 was considered significant.

Values in the same row with different superscript letters are significantly different.

Significant values are in bold.

### The interaction between MC4R rs17782313 and CSI and N6/N3 on metabolically unhealthy phenotype

Using the GLM, we found no association between MC4R rs17782313 polymorphism and CSI on MUH phenotype in a multivariate-adjusted model controlling for the covariates. But the CC genotype of MC4R rs17782313 interacts with the N6/N3 ratio on the metabolically unhealthy phenotype. We found that interaction between CC genotype and N6/N3 on metabolically unhealthy phenotype in the crude model (β = 9.94, CI 2.49–17.39, *P* = 0.009) and even after adjustment for all confounders (β = 9.002, CI 1.15–16.85, *P* = 0.02, β =  − 12.12, CI 2.79–21.46, *P* = 0.01), This means that those who homozygously have the risk allele as CC genotype, are more likely to have an unhealthy phenotype with an increase in N6/N3 than those who do not have CC genotype (Table 4).

**Table 4:** The interaction between MC4R rs17782313 and CSI and N6/N3 on metabolically unhealthy phenotype.

Variable	MC4R		Crude			Model 1			Model 2		
			B	CI	P	B	CI	P	B	CI	P
**Phenotype**	**CSI**										
Metabolic unhealthy	TT	Reference									
	TC		0.013	− 0.18 to 0.20	0.89	0.40	− 0.15 to 0.24	0.69	0.49	− 0.15 to 0.25	0.63
	CC		− 0.97	− 0.26 to 0.074	0.26	− 0.10	− 0.28 to 0.07	0.23	− 0.11	− 0.29 to 0.06	0.21
**Phenotype**	**N6/N3**										
Metabolic unhealthy	TT	Reference									
	TC		4.001	− 3.22 to 11.22	0.27	0.90	− 6.62 to 8.43	0.81	1.37	− 6.62 to 9.38	0.73
	CC		9.94	2.49 to 17.39	**0.009**	9.002	1.15 to 16.85	**0.02**	12.12	2.79 to 21.46	**0.01**

GLM was performed to identify the interaction between MC4R rs17782313MC4R and CSI and N6/N3 on metabolic unhealthy phenotype Model 1 = adjusted for potential confounding factors including (age and BMI).

Model 2 = adjusted for potential confounding factors including (age, BMI, energy and physical activity).

*p* < 0.05 was considered sig.

Significant values are in bold.

## Discussion

This study showed those who have the CC genotype are more likely to have an unhealthy obesity phenotype with higher N6/N3 ratio than those who have the CC genotype with lower intake of N6/N3 ratio.

This cross-sectional study reported that according to tertiles of fat quality indices, a higher HOMA IR index was accompanied by higher ratio of N6/N3. Among the genotypes of 279 obese women with the MC4R gene, higher CSI was linked with more refined grain, vegetables, fish, poultry, egg, red meat, protein, carbohydrate, total fat, cholesterol, PUFA, SFA, linolenic acid, EPA, and DHA. Across the higher tertile of N6/N3, we found lower consumption of carbohydrates, MUFA, PUFA, oleic acid, and linoleic acid.

A multivariate-adjusted model controlling for covariates found no interaction between the MC4R rs17782313 polymorphism and CSI on the MUH phenotype. But the CC genotype of MC4R rs17782313 interacts with the N6/N3 ratio on the metabolically unhealthy phenotype. Most previous studies support our result. For example, C allele carriers significantly had higher total cholesterol and TG levels in comparison to the TT genotype of MC4R in Brazilian obese children and adolescents, which can cause dyslipidemia^46^. It was additionally established that the risk of diabetes in carriers of the C allele increased by 14% regardless of the BMI. While another study showed that total cholesterol and LDL were not associated with different genotypes of MC4R^47,48^. Some other studies explain the interaction of the CC genotype of MC4R with the N6/N3 ratio on the metabolically unhealthy phenotype as follows: one paper expressed that body weight and BMI were higher in the CC and CT groups compared with individuals in the TT group, which can cause an unhealthy phenotype^49^; another reported the presence of the C allele in Mexican adults can have a possible influence on increasing fasting glucose levels^48,50^; but the CC genotype in European, is just associated with higher BMI and waist, but not with other variables of metabolic disorder^51^; another one a found higher prevalence of hyperglycemia and diabetes in women with the CT/CC genotype and explained this association by the higher obesity prevalence in this group^52^. Aside from fat tissue size, the distribution of body fat accumulation, whether subcutaneous Aside from fat tissue size, the distribution of body fat accumulation, whether subcutaneous or viscerally, is more important^46^ because visceral adipocytes are more metabolically active and can lead to the development of insulin resistance (IR) and all-cause mortality.us or viscerally, is more important^46^ because visceral adipocytes are more metabolically active and can lead to the development of insulin resistance (IR) and all-cause mortality^53–55^. Moreover, higher visceral adipose tissue (VAT)/subcutaneous adipose tissue (SAT) ratios may be associated with increased metabolic and cardiovascular risk, independently of BMI and VAT content^56^. We found in our study that CC genotype is associated with metabolically unhealthy situations regardless of BMI or visceral fat level, and this may happen because of lower height or higher HOMA index in C allele carriers.

In our study, a higher intake of the ratio of N6/N3 was associated with a higher HOMA IR index. As a result, two studies expressed that a higher N6/N3 ratio has a worse effect on HOMA-IR and quantitative insulin-sensitivity check index (QUICKI) indices^57,58^. In addition, an article reported that type 1 diabetes (DT1) was positively correlated with foods rich in PUFAs^28^ But in contrast to our result, another study found no effect of flax seed on HOMA-IR, although the N6/N3 ratio was lower in this group^59^. Some other studies examined the benefits of lower intake of N6/N3 ratio, failed to show a significant effect on insulin sensitivity^60^ the result we found is because of the effect of higher N6/N3 ratio on the expression of inflammatory markers^57^ which can induce HOMA-insulin resistance^61^.

Participants with the C allele had lower height and higher HOMA index. Our finding in this regard was in line with previous observational studies^48^ another study found that males with CC genotype had higher serum glucose levels compared with the other genotypes (TT and TC), the study expressed that C-allele carriers in the rs17782313 have an increased susceptibility to MUHO compared to the T-allele carriers^20^. T Schritter et al. demonstrated that in subjects carrying the C allele homozygous or the heterozygous form, the insulin response reduced as compared to TT genotype^21^. But no statistically significant difference in HOMA index and height status across MC4R rs17782313 genotypes among women and men was seen in some studies^62^.

Higher CSI was linked with more consumption of refined grain, vegetable, fish, poultry, egg, red meat, protein, carbohydrate, total fat, cholesterol, PUFA, SFA, linolenic acid, EPA, and DHA, and higher tertile of N6/N3 associate with lower intake of carbohydrate, MUFA, PUFA, oleic acid, and linoleic acid. According to previous studies higher intake of protein especially animal protein associated with higher cholesterol and SFA intake which can cause higher CSI index^63,64^ We know that consuming foods that are rich in omega 6 and have higher ratio of N6/N3, accompanied with lower intake of MUFA, omega 3 and oleic acid, and this can induce inflammation^65^. It is important to mention the strengths of the present study, first, was its novelty, to our knowledge it is the first study that investigated the interaction of dietary fat quality indices and the MC4R gene in metabolically healthy and unhealthy overweight and obese women. Other strengths are that we used validated FFQ questionnaires based on the Iranian population. As our strengths, there are some limitations to our study, first the cross-sectional design of our study, ruling out any causal relationship. The second limitation is the reliance on self-reported dietary data, which can cause information bias. Since our study only included overweight and obese women, we cannot generalize the results of this study to all women in the population.

## Conclusion

The present study explains the interaction between genetics and the environment. Higher ratio intake of N6/N3 in CC genotype associates with unhealthy phenotype. In addition, C allele carrier of MC4R showed lower height and higher HOMA index. These results can help us to have better dietary recommendations about metabolic health status.

## Data Availability

The datasets generated and/or analyzed during the current study are not publicly available due to the restrictions and the research rules of the Tehran University of Medical Sciences (TUMS) but are available from the corresponding author on reasonable request.

## Abbreviations

ANCOVA: Analysis of covariance
ANOVA: Analysis of variance
BF: Body fat
BIA: Bioelectrical impedance analyser
BMI: Body mass index
CRP: C-reactive protein
CSI: Cholesterol-saturated fat index
DHA: Docosahexaenoic acid
DNA: Deoxyribonucleic acid
EPA: Eicosapentaenoic acid
FFM: Fat free mass
FFQ: Food frequency questionnaire
GLM: Generalized linear model
GPOPAP: Glycerol-3-phosphate oxidase–phenol 4-amino antipyrine peroxidase
HDL: High-density lipoprotein
HOMA-IR: Homeostatic model assessment for insulin resistance
HR: Hip circumference
HS-CRP: High sensitive C-reactive protein
IPAQ: International physical activity questionnaire
IRX3: Iroquois homeobox protein 3
LDL: Low-density lipoprotein
MET: Metabolic equivalent
MC4R: Melanocortin 4 receptor
MHO: Metabolically healthy obese
MUFA: Monounsaturated fatty acid
MUO: Metabolically unhealthy obese
N6/N3: Omega-6/omega-3
PA: Physical activity
PCR-RFLP: Polymerase chain reaction-restriction fragment length polymorphism
PUFA: Polyunsaturated fatty acid
QUICKI: Quantitative insulin-sensitivity check index
RPGRIP1L: Retinitis pigmentosa GTPase regulator-interacting protein 1 like
SD: Standard deviation
SFA: Saturated fatty acid
SAT: Subcutaneous adipose tissue
SNPs: Single-nucleotide polymorphisms
TG: Triglyceride
VAT: Visceral adipose tissue
WC: Waist circumference
WHR: Waist to hip ratio
